# Application of the Delphi Method in the Construction of an Evaluating and Grading Scale for Evidence of Disease Prevention and Treatment in Ancient Books of Traditional Chinese Medicine

**DOI:** 10.1155/2022/3674663

**Published:** 2022-03-01

**Authors:** Lei Zhang, Xinfeng Guo, Shuo Yang, Sihong Liu, Hongtao Li, Guangkun Chen, Hongjie Gao, Huamin Zhang, Lin Tong

**Affiliations:** ^1^Institute of Information on Traditional Chinese Medicine, China Academy of Chinese Medical Sciences, No. 16, Dongzhimen South Alley, Dongcheng District, Beijing 100700, China; ^2^The Second Clinical College of Guangzhou University of Chinese Medicine, No. 111, Dade Road, Yuexiu District, Guangzhou 510120, China; ^3^Institute of Chinese Materia Medica, China Academy of Chinese Medical Sciences, No. 16, Dongzhimen South Alley, Dongcheng District, Beijing 100700, China

## Abstract

**Objective:**

This study aimed to develop a scale for evaluating and grading the evidence of prevention and treatment in ancient books of traditional Chinese medicine (TCM), in view of providing a reference for TCM clinicians, supporting the compilation or revision of evidence-based practice guidelines for TCM, improving the level of evidence-based research on ancient TCM books, and supplementing the development of evidence-based ancient TCM books.

**Methods:**

The Delphi method was used for consultation among 40 experts in relevant fields. Excel 2016 and SPSS 21.0 were used to analyze the positive coefficient, authority coefficient, degree of concentration, degree of coordination, and degree of expert consensus.

**Results:**

In the first round of the questionnaire, a total of 17 evaluation indexes were formulated in three aspects: 5 were deleted, 1 was modified according to the expert opinions, and no additional index was added. In addition, quantitative standards, weight assignment, and grading standards were developed according to the findings of the expert consultation. The positive coefficients of experts in the first and second rounds of questionnaires were 87.5% and 82.9%, respectively. The authority coefficient was 0.835 (>0.7). The coefficient of variation of the first and second rounds were 0.14∼0.29 and 0.09∼0.27, respectively. Kendall's coefficient of concordance of the first and second rounds were 0.135 (*p* < 0.05) and 0.081 (*p* < 0.05), respectively.

**Conclusion:**

The evaluation indexes and quantitative reference values of the developed scale conform to the characteristics of prevention and treatment evidence in ancient TCM books. It can provide a useful exploration tool for the evaluation and grading of evidences in TCM ancient books.

## 1. Introduction

Ancient books of traditional Chinese medicine (TCM) compiled and published in 1911 and before are the only books to carry the text on hand-made paper and are important and direct carriers of TCM knowledge. Among them, the contents of ancient books on prevention and treatment, which are known to have very important practical value, have been repeatedly verified by physicians in the past dynasties in clinical practice. With the rapid development of evidence-based medicine, evidence plays an increasingly important role in medical practice. The ancient books of TCM are the main source of evidence for the treatments in TCM clinical medicine. Currently, there are no relevant standards for evidence-based research in evidence quality evaluation and classification and the current research status in this field is still being explored [1]. For instance, Liu et al. suggested that expert consultation should be adopted to screen the objective evaluation indicators of evidence in ancient books [2, 3]. Ren et al. proposed five evaluation indexes to evaluate the quality of evidence, including the reliability of evidence source, application intensity, medical qualification, nature of evidence content, and integrity of evidence content, in the construction of an acupuncture evidence evaluation system [4]. Li et al. believed that the evaluation of the evidence quality of ancient books should be carried out from three perspectives, including patient self-evaluation, medical expert evaluation, and other evaluation out [5]. Wang believed that the basis of evidence evaluation about ancient books was mainly to analyze the creativity and citation rate of knowledge in ancient books and comprehensively complete the evaluation of knowledge by combining elements, such as the degree of “other evaluation and self-evaluation” of later generations [6]. At the same time, the evaluation of TCM ancient books is inseparable from the evaluation of ancient books and doctors, which generally constitutes a “three-dimensional integrated” framework system. In terms of quality of evidence classification, the research result of Liu is more recognized [7]. The classification standard refers to the method in modern evidence-based medical evidence classification, which covers the modern clinical research of TCM, the Chinese medical evidence classification of evidence, and the standard was updated. The updated version highlighted in ancient evidence of TCM and modern clinical research evidence due to differences in many aspects, such as content and style, it is inappropriate to apply the same scale of measurement [7]. Therefore, the new version is mainly aimed at the modern clinical research of the TCM classification standard for the development of evidence. The classification of modern clinical research evidence and the criteria for escalation or degradation were also described in detail.

Based on the abovementioned problems and the present situation, to develop effective evaluation system for Chinese medical evidence has a wide range of applications and meets the demand for “best evidence” of evidence-based medicine. This study draws lessons from the current research of evidence-based practice in classifying the evidence and characteristics of classification, considering the particularity of ancient literature of TCM in writing style and content of the report, to develop an evaluating and grading scale for the prevention and treatment evidence of ancient Chinese medical books, in order to provide a reference for clinical physicians and medical researchers to participate in the formulation of TCM clinical practice guidelines [8].

## 2. Materials and Methods

### 2.1. Establishment of a Pool of Evaluation Entries

What elements should be included in the establishment of an evidence evaluation index system of ancient books of TCM? This study first investigated the related research by retrieving information from China National Knowledge Infrastructure (CNKI), Wanfang Data, China Science and Technology Journal Database (VIP), and Chinese biomedical database (CBM) using the search terms “Ancient books of TCM” OR “Classical medical literature for medicines” OR “Ancient literature of TCM” AND (“Evidence-based medicine” OR “Evidence”). It aimed to analyze the method of evaluating the quality of TCM evidence in ancient books in modern literature. But on account of the particularity of content and style in TCM ancient books and in order to get the comprehensive evaluation index system in this study, we selected the *Synopsis of the Golden Chamber* (*Jin Gui Yao Lve*), which is a classic in internal medicine with miscellaneous diseases and theories. *Formularies of the Bureau of People's Welfare Pharmacy* (*Taiping Huimin Hejiju Fang*), which is the world's first large-scale formula book compiled by the official auspices. *Treatise on the Spleen and Stomach* (*Piwei Lun*), which is a representative work of one of the four famous medical scientists in the *Jin* and *Yuan* dynasties, *and Classified Case Records of Celebrated Physicians* (*Mingyi Leian*), which is the foundation work of TCM medical cases. Meanwhile, we browsed extensively the record forms and characteristics of various works in the *Encyclopedia of Traditional Chinese Medicine (Zhong Hua Yi Dian,* Version 5), which is the major TCM work of all dynasties with 1,156 ancient Chinese medical books, totaling over 10,000 volumes and more than 400 million words before 1949 [9, 10]. In view of the abovementioned contents, entries related to evaluation indexes were extracted, and a pool of evaluation entries of ancient Chinese medicine books evidence was developed.

### 2.2. Questionnaire Design

The questionnaire was designed mainly based on the pool of evaluation entries established. The content of the questionnaire includes a letter to the experts, which explains the purpose, content, and method of expert consultation in brief. Subject of the questionnaire, which is an expert evaluation score table set the importance score for each index of the evaluation index of evidence of ancient books, experts are invited to assign the importance of the indexes according to the Likert 5-level scoring method (very important, important, generally important, not too important, and not important are 5, 4, 3, 2, and 1 point, respectively), and a column of suggestions for modification is attached for experts to put forward suggestions and reasons for modification, addition, or reduction. Basic information of experts, including the general information of experts, the evaluation of experts' judgment on the consulting content, and the evaluation of experts' familiarity with the consulting content are included. The judgment basis includes four dimensions: practical experience, theoretical analysis, peer understanding, and intuitive selection. The influence degree of expert judgment of each dimension is divided into three grades: large, medium, and small. Those are practical experience (0.5, 0.4, and 0.3), theoretical analysis (0.3, 0.2, and 0.1), peer understanding (0.1, 0.1, and 0.1), and intuitive selection (0.1, 0.1, and 0.1). The familiarity degree is divided into five grades, including very familiar, relatively familiar, general familiar, not very familiar, and not familiar, and the values are 1.0, 0.8, 0.5, 0.2, and 0 in sequence.

### 2.3. Expert Selection and Consultation

40 experts were invited from across China for consultation. The selection criteria of experts were as follows: experts had a deputy senior professional title or above, engaged in TCM clinical practice, TCM ancient literature research and TCM research, had a rigorous academic attitude, showed some enthusiasm for our research, and were willing to answer the expert consultation questionnaire. We sent questionnaires by expressage, and prior to this, the experts were informed about the main purpose, method, and time schedule of this study via phone calls or SMS, so that the experts could fully understand the intended purpose of the questionnaire and offer their support.

### 2.4. Statistics Analysis

The results of the questionnaire were checked and entered in Excel 2016 and SPSS 21.0 by two researchers. According to the Delphi method, the expert positive coefficient, that is, the ratio between the number of returned questionnaires and the total number of sent questionnaires [11]. The authority coefficient (represented by Cr), based on familiarity with the contents of the questionnaire (represented by Cs) and judgment coefficient (represented by Ca), the specific calculation method is Cr = (Cs + Ca)]/2 [12]. The degree of concentration of expert opinions is expressed by the arithmetic mean and full mark ratio of the importance score given by the consulting experts for each evaluation index. The degree of coordination is used to reflect the degree of consistency of experts' opinions on the evaluation indicators in the questionnaire. It is usually reflected by the coefficient of variation (CV) and Kendall's coefficient of concordance (Kendall's W). The CV reflects the degree of dispersion of expert opinion [13], Kendall's W valuates the degree of coordination among experts on the evaluation object. Its value is between 0 and 1, and the higher the value represents the consistency of expert opinion, the better [14].

### 2.5. Determine the Weight of Each Indicator

Apply the expert consultation method of the subjective weighting method to determine the weight of each index; that is, experts judge the importance of the index and normalize it according to the average importance degree of each index. The basic principle of the method is that more important indicators should be given a larger weight. The weight of each indicator is judged by experts according to their own experience and subjective judgment of the actual situation.

## 3. Results

### 3.1. Results of the Establishment of a Pool of Evaluation Entries

The results of literature survey are shown in the Table 1. Through the literature review, it was found that the content of evidence is a factor affecting its quality. The classification of the content of the evidence should be the first step for evidence evaluation, which is consistent with modern evidence-based practice. In this study, the evidence in ancient TCM books was divided into the following two categories: evidence of knowledge and evidence of case. The evidence of knowledge refers to the evidence recorded in canons, medical classics, formula books, and clinical specialty books that mainly expound on theoretical viewpoints without clinical practices, or those summarized as the evidence in ancient books other than that in medical cases and medical notes. Ancient books that record evidence of knowledge are called ancient books of knowledge. The evidence of cases refers to those recorded in clinical case recordings, such as medical cases and medical notes, and those about empirical experience. The ancient books that record case evidence are called ancient books of cases.

### 3.2. Positive Coefficient

In the first round of expert consultation, a total of 40 questionnaires were sent out to experts, and 35 were recovered, with a recovery rate of 87.5%. In the second round, a total of 35 questionnaires were issued, and 29 were recovered, with a recovery rate of 82.9%. The recovery rate of each round of questionnaires was higher than 70%, indicating that the experts participating in this study were highly motivated and cooperated well with the study team.

### 3.3. Authority Coefficient

In our study, the value of Cs is 0.83, the value of Ca is 0.84, and the value of Cr is 0.835 by formula, which is bigger than 0.7, indicating that the experts consulted have high authority and can improve the reliability of the research.

### 3.4. Concentration of Expert Opinions

Results of the full mark ratio and the arithmetic mean of each evaluation index in the two rounds of questionnaires are shown in Table 2. The full mark of some items was relatively small, indicating that the concentration degrees of such items were poor.

### 3.5. Degree of Expert Coordination

The results of the CV of each evaluation index in the two rounds of questionnaires are shown in Table 2. The CV of the first round fluctuated between 0.14 and 0.29. The evaluation indexes with poor consistency are A2 (quantity of citing others), A3 (book written time), and A15 (sample size of treatment). The CV of the second round fluctuated between 0.09 and 0.27, and the evaluation index with poor consistency was A4 (number of editions), and so on.

Results of Kendall's coefficient of concordance are shown in Table 3. In the first round, Wi was 0.135 (*p* < 0.05). In the second round, Wi was 0.081(*p* < 0.05) and the difference was statistically significant, indicating that the experts participating in the two rounds had consistent opinions on the evaluation indicators.

### 3.6. Results of Expert Consultation on Evaluation Indexes

The results of the calculation of the importance score and consensus degree of each evaluation index are shown in Table 4. Based on the inclusion criteria of the evaluation indexes of this study, the results are as follows: 2 indexes (A2 and A3) were deleted from the evaluation of ancient books where the evidence came from; 3 indexes (A10, A14, and A15) were deleted from the evaluation of case evidence, and no new ones were added. A2, which is an index on source ancient books and a score on experts' consensus and importance, met the inclusion criteria; however, several experts suggested its exclusion from the note column, as they believe that this index has poor operability due to the characteristics of TCM ancient books. From the perspective of the digitization degree and scale of ancient books, it can be difficult to quantitatively measure how one ancient book cited others; therefore, it is difficult to obtain a result. Therefore, the experts' opinions were accepted, and A2 was excluded. Finally, a total of 12 evaluation indexes were included in the evaluation index list, including three for ancient book sources, four for knowledge evidence, and five for case evidence. At the same time, A5 (source of ancient books) was revised to A5 (popularity of ancient books) based on the experts' opinions.

### 3.7. Determination of Quantitative Methods of Evaluating Indicators in the Scale

The second round of expert questionnaire survey is mainly about consultation on the quantitative methods of each evaluation index. According to the different contents of the evaluation index, qualitative and quantitative methods were adopted. The qualitative method is a common method to score the relevant evaluation items according to the qualitative description. For example, the AMSTAR scale is a scale to evaluate the quality of systematic evaluation research. The evaluation result of each evaluation index of this scale is a qualitative result of “yes” or “no”. Jin et al. [15] applied the AMSTAR scale by assigning 1 score to “yes” and 0 score to “no.” This study used the method for reference to quantify the relevant indicators, such as “Is the content of disease prevention and treatment comprehensive? Overall is scored 5 points, basic overall is scored 3 points, if it is not comprehensive, it will be 1 point.”

The quantitative method is to score the relevant evaluation items according to the quantitative description. In this study, the method of pre-evaluation was adopted, and the treatment formulae selected from the “TCM Clinical Practice Guide for Chronic stable Angina pectoris” in the TCM Evidence-based Clinical Practice Guide: Internal Medicine of TCM [16] were used as evaluation objects, and relevant evaluation indicators were used for pre-evaluation. The mean and median values of 10 interventions for each evaluation index were calculated, and the results are shown in Table 5. Due to the extreme value of the obtained data compared with the mean, statistical experts were consulted to select the median as the middle value to set the quantitative reference value. The specific method was 1 point for the minimum value of the pre-evaluation result, 3 points for the median value, and 5 points for the maximum value. For example, “How did other ancient books of knowledge study this evidence? More than 1300 items count as 5 points, 500–1300 items count as 3 points, 10–500 items count as 1 point.”

According to the results of the second round, the consensus degree was calculated, which indicated that the experts' consensus degree on all evaluation indexes was above 60%. In the experts' opinion and suggestion session, some experts believe that in the *General Catalogue of Ancient Chinese Medical Books* (Zhongguo Zhongyi Guji Zongmu), most ancient books have two to three versions and only a few have dozens of editions. Therefore, it is unreasonable to quantitatively measure the number of editions (suggestions are shown in Appendix 1). Appendix 1 also shows the second round's questionnaire body content and the experts' consensus degrees, opinions on the treatment, and modification conditions of the evaluation indicators.

### 3.8. Determination of Relevant Weights in the Scale

According to the results of the second round of expert questionnaire, the weight ratio between the ancient books and the evidence content was 3 : 7. The subjective weighting method was applied to calculate the weight value of each evaluation index, and normalized meanwhile. The sum of each weight value was 1, and the results are shown in Appendix 1. In addition, for the convenience of calculation, all results were multiplied by 10 and approximated values were used. For example, 1.9 is adjusted to 2, while 3.4 is adjusted to 3.5. The specific results are shown in Appendix 1.

### 3.9. Establishment of Grading Standards

According to the preliminary formation of the scale, different weights of the evaluation indexes, score arrangements of evidence's content (evidence of knowledge or evidence of case), and the total score were evaluated. The grading standards are as follows with advice from the consulted experts in the questionnaires: high quality evidence: 35 points or above; intermediate quality evidence: 20 points or above; and low quality evidence: less than 20 points (Note: For one prescription, if it is used both as evidence of knowledge and case, the prescription will be promoted to a higher level on its original classification results according to the evaluation standard of knowledge evidence and will not be evaluated in the evidence of a case session. As for one case-based evidence, all will be included at first, but the final evidence level accords with the highest level.).

### 3.10. Form and Formation of the Scale

The final evaluation rating scale consists of three main parts: the body of the scale, the rating standard for each index, and the grading standard for evidence quality. Specific quantitative measures and operations are illustrated in the rating standard in detail (Appendix 2). When evaluating the popularity of ancient books, this study sorted out an evaluation reference material according to the *History of Chinese Medicine*[17] in order to provide a reference for the users of this scale.

### 3.11. Application of Scale in Knee Osteoarthritis (KOA)

With reference to Zhongyi Neike Xue (Internal Medicine of Traditional Chinese Medicine) [18], Zhongyi Da Cidian (Dictionary of Traditional Chinese Medicine) [19], TCM monographs on osteopathy [20–22], and clinical guidelines [23] for names and symptoms of KOA in TCM, the search term was determined, including *“bi zheng,” “bi,” “bi disease,” “gu bi,” “he xi feng,” “li jie,” “wang bi,” “wan bi,” “knee bi,” “li jie feng,” “li jie disease,” “jin bi,” “white tiger disease,” “knee pain,”* and *“tong feng.”* We use the abovementioned search terms to obtain evidence from the *Encyclopedia of Traditional Chinese Medicine (Zhong Hua Yi Dian,* version 5).

This study sorted out the evidence contents of ancient Chinese medicine books for the treatment of KOA referring to the studies of May et al. [24–27], who have done a good standardized demonstration on the evidence arrangement of ancient Chinese medicine books in terms of evidence collation. Applying our scale to evaluate and grade the evidence, a total of 141 pieces of evidence of the treatment of knee osteoarthritis in ancient Chinese medicine books were selected finally. Buzhong Yiqi decoction (plus or minus) was the high level evidence, and the 11 evidence, such as Siwu (plus or minus), Wuji powder (plus or minus), Shiquan Dabu decoction (plus or minus), and Dafangfeng decoction, were the middle level evidence. The 128 pieces of evidence of Liuwei pill, Yunmu cream, Touguan powder, and Chuanmutong decoction were of low grade (Table 6). At present, the TCM clinical practice guidelines for KOA includes Juanbi decoction, Simiao decoction, Taohong Siwu decoction, Duhuo Jisheng decoction, and Bazhen decoction [28]. According to the results of this study, a variety of other prescriptions can be recommended for the treatment of KOA.

## 4. Discussion

Since ancient times, the ultimate goal of all medical research has been to save lives and improve the clinical efficacy of diseases. The origin and development of evidence-based medicine to clinical diagnosis and treatment with a focus on the process of using evidence to clinical curative effect, more specifically, the application of the evidence-based medicine method to systematically find evidence, screening, and evaluation of evidence and combining it with the experience of the patients and clinicians using evidence to make up for the lack of medical experience, is considered to be an irreplaceable scientific method in clinical practice and clinical decision. As an important part and source of TCM evidence, the idea and method of evidence-based medicine are applied to establish a perfect evaluation system of TCM evidence, so as to improve the reliability and scientificity of TCM clinical research and promote the application of TCM to the world.

We first applied the Delphi method to construct an evaluating and grading scale for evidence of disease prevention and treatment in ancient books of TCM based on sufficient literature investigation under the guidance of theory and practice of evidence-based medicine. The evidence from the ancient books of TCM is divided into knowledge evidence and case evidence, and every kind of evidence was evaluated from both the source of evidence and the content of evidence separately. In terms of the evaluation of evidence sources, this study evaluates ancient Chinese medicine books from three different perspectives; they are the number of citations, the number of versions, and the popularity of ancient Chinese medicine books, which play an important role in ensuring the source reliability of evidence in ancient Chinese medicine books. Li et al. also believed that compared with modern evidence-based evaluation, the evaluation of evidence in ancient books should focus more on which ancient book the evidence is from [5]. In terms of the evaluation of evidence content, this study divides evidence into knowledge evidence and case evidence according to the different nature of the content to establish evaluation indicators, respectively. In the aspect of knowledge evidence evaluation, whether the description of evidence content is comprehensive should be regarded as an indicator to evaluate the quality of evidence. If the relevant contents of disease prevention and treatment are described completely and comprehensively, the evidence users can obtain the overall idea and measures of preventing and treating disease and apply the evidence in the clinical practice of traditional Chinese medicine. The situation that ancient book evidence is studied in other ancient books of knowledge, ancient books of cases, and modern literature is the concrete embodiment of the application intensity of evidence, which can reflect the extensive application of ancient book evidence, so application of ancient books evidence in other medical literature can be used as the evaluation index to distinguish the quality of ancient book evidence. Case evidence mainly refers to clinical case records or empirical evidence such as medical cases and sayings. There is an obvious difference between case evidence and knowledge evidence, either in content or in writing style. Case evidence is a full representation of the process of clinical diagnosis and syndrome differentiation of an ancient doctor, contains the patient's personal basic information, specific diagnosis, treatment of disease, and clinical curative effect, and so on. We mainly establish an evaluation index according to the evaluation of its characteristics.

Finally, an evaluation grading scale containing 12 evaluation indexes is formed, which includes the specific quantitative score, weight assignment, and grading standard of each evaluation index. At the same time, a detailed description of each scoring standard of the scale is attached at the end of the whole scale. The scale made a specific quantitative evaluation method and the classification standard for ancient books' evidence. It has a supporting role both in the clinical application of TCM and in developing or revising the TCM clinical evidence-based practice guidelines. The scale makes an appropriate supplement for evaluation and recommendation of evidence of ancient TCM books.

The following specific problems should be paid attention to in the application of the scale: First is to determine the search term. In modern clinical medicine, a disease may correspond to a variety of diseases of TCM. We should correspond to the names of diseases in western medicine, Chinese medicine, and the names of diseases in TCM ancient books, search for information comprehensively, and determine the search terms as comprehensive as possible, so as to make the retrieved evidence more comprehensive. Another problem is to set out the inclusion and exclusion criteria for evidence. The inclusion and exclusion criteria for evidence in TCM ancient books should be specific and clear in their formulation. The two criteria should be complementary rather than contradictory, and the criteria should be adjusted at any time according to the target disease.

## 5. Conclusion

The evaluation indexes and quantitative reference values of the developed scale conform to the characteristics of prevention and treatment evidence in ancient TCM books. It can provide a useful exploration tool for the evaluation and grading method of TCM ancient book evidence.

## Figures and Tables

**Table 1:** Summaries of evaluation indexes or influencing factors of the quality of TCM ancient books from the literature.

No.	Evaluation indexes or influencing factors
1	Character of the evidence (theories or cases)
2	Reliability of origin
3	Qualification of the clinic doctors
4	The title of an ancient book
5	The creator of an ancient book
6	Origin of the ancient books
7	Citation frequency
8	Citation amount
9	Ratio of being cited
10	Edition amount
11	Completeness (comprehensive response in etiology, pathogenesis, treatment program, and efficacy)
12	Strength of application of the evidence

**Table 2:** Experts' concentration level and variation coefficient results of the first round.

Classification	Evaluation indicator	Full mark ratio		Arithmetic mean		Standard deviation		Coefficient of variation	
		First round	Second round	First round	Second round	First round	Second round	First round	Second round
Evidence's source (ancient book)	(1) Quantity of being cited (A1)	0.68	0.86	4.54	2.72	0.92	0.70	0.20	0.26
	(2) Quantity of citing others (A2)	0.29	0.69	3.86	2.52	1.03	0.69	0.27	0.27
	(3) Book written time (A3)	0.31	0.90	3.86	2.90	1.12	0.31	0.29	0.11
	(4) Quantity of version (A4)	0.30	0.86	3.97	2.83	1.04	0.47	0.26	0.17
	(5) Source of ancient books (A5)	0.57	0.79	4.51	2.72	0.61	0.59	0.14	0.22

Evidence of knowledge	(1) Is the description of disease treatment comprehensive? (A6)	0.54	0.83	4.40	2.76	0.74	0.58	0.17	0.21
	(2) Are they extensively studied in other ancient medical books of knowledge? (A7)	0.29	0.72	4.14	2.66	0.65	0.61	0.16	0.23
	(3) Is it widely used in medical cases and notes? (A8)	0.49	0.90	4.29	2.93	0.79	0.26	0.18	0.09
	(4) Is it widely used in modern literature? (A9)	0.34	0.86	4.09	2.86	0.82	0.35	0.20	0.12

Evidence of case	(1) Is the patient's personal information comprehensive? (A10)	0.23	0.76	3.83	2.72	0.86	0.53	0.22	0.19
	(2) Is diagnosis and treatment information comprehensive? (A11)	0.71	0.86	4.63	2.86	0.65	0.44	0.14	0.15
	(3) Is the number of visits for disease treatment reported? (A12)	0.40	0.79	4.17	2.76	0.89	0.51	0.21	0.19
	(4) Is efficacy reported? (A13)	0.71	0.86	4.54	2.72	0.78	0.70	0.17	0.26
	(5) Is follow-up information reported? (A14)	0.31	0.69	3.91	2.52	0.98	0.69	0.25	0.27
	(6) Sample size of treatment? (A15)	0.34	0.90	3.89	2.90	1.02	0.31	0.26	0.11
	(7) Notes or explanation on diagnosis and treatment gist and thinking? (A16)	0.46	0.86	4.29	2.83	0.75	0.47	0.18	0.17
	(8) Is it widely used in modern literature? (A17)	0.34	0.79	4.17	2.72	0.71	0.59	0.17	0.22

**Table 3:** Results of the first two rounds' coordination of experts' opinions.

	First round	Second round
*N*	35	29
Kendall W^a^	0.135	0.081
Chi-square	75.406	25.790
Df	16	11
Asymptotic significance	0.000	0.007

^a^Kendall.

**Table 4:** Results of the first round of expert questionnaire survey.

Classification	Evaluation indicator	Importance score	Consensus (%)	Result	Modification
Evidence's source (ancient book)	(1) Quantity of being cited (A1)	4.54	100.00	Included	No
	(2) Quantity of citing others (A2)	3.26	74.29	Deleted	
	(3) Book written time (A3)	2.91	65.71	Deleted	
	(4) Quantity of version (A4)	3.40	77.14	Included	No
	(5) Source of ancient books (A5)	4.34	94.29	Included	Popularity of the ancient books

Evidence of knowledge	(1) Is the description of disease treatment comprehensive? (A6)	3.97	85.71	Included	No
	(2) Are they extensively studied in other ancient medical books of knowledge? (A7)	3.71	85.71	Included	No
	(3) Is it widely used in medical cases and notes? (A8)	3.69	80.00	Included	No
	(4) Is it widely used in modern literature? (A9)	3.43	77.14	Included	No

Evidence of case	(1) Is the patient's personal information comprehensive? (A10)	2.86	65.71	Deleted	
	(2) Is diagnosis and treatment information comprehensive? (A11)	4.37	91.43	Included	No
	(3) Is the number of visits for disease treatment reported? (A12)	3.83	85.71	Included	No
	(4) Is efficacy reported? (A13)	4.37	91.43	Included	No
	(5) Is follow-up information reported? (A14)	2.94	65.71	Deleted	
	(6) Sample size of treatment? (A15)	2.97	65.71	Deleted	
	(7) Notes or explanation on diagnosis and treatment gist and thinking? (A16)	3.77	82.86	Included	No
	(8) Is it widely used in modern literature? (A17)	3.66	82.86	Included	No

**Table 5:** Evaluation results of 10 “pre-evaluation” prescriptions.

Prescriptions name	Ancient books	Quantity of version	Quantity of being cited	Are they extensively studied in other ancient medical books of knowledge?	Is it widely used in medical cases and notes?	Is it widely used in modern literature?
Gualou Xiebai Banxia decoction	*Jin Gui Yao Lve*	31	1878	85	6	18220
Shengmai powder	*Nei Wai Shang Bian Huo Lun*	13	28	1256	251	9320
Shenfu decoction	*Shi Yi De Xiao Fang*	6	130	482	55	5720
Xuefu Zhuyu decoction	*Yi Lin Gai Cuo*	51	128	37	2	37400
Baoyuan decoction	*Bo Ai Xin Jian*	6	37	1266	90	1720
Suhexiang pill	*Taiping Huimin Hejiju Fang*	30	191	1103	95	743
Dangui Sini decoction	*Shang Han Lun*	27	5304	664	52	19700
Danshen yin	*Shi Fang Ge Kuo*	33	28	12	5	6160
Zhishi Xiebai Guizhi decoction	*Jin Gui Yao Lve*	31	1878	96	6	2580
Zhigancao decoction	*Shang Han Lun*	27	5304	532	74	5820
*Mean*		25.5	1490.6	553.3	63.6	10738.3
*Median*		28.5	160	507	53.5	5990

**Table 6:** Evaluation results of ancient book evidence for KOA.

Evidence	Level	Source
Buzhong Yiqi decoction (plus or minus)	High	*Shou Shi Bao Yuan*
Siwu decocotion (plus or minus)	Middle	*Wan Bing Hui Chun*
Wuji powders (plus or minus)	Middle	*Wan Bing Hui Chun*
Shiquan Dabu decoction (plus or minus)	Middle	*Shou Shi Bao Yuan*
Dafangfeng decoction (plus or minus)	Middle	*Chi Shui Xuan Zhu*
Fuling decoction	Middle	*Bao Ming Ge Jue*
Qingzao decoction	Middle	*Shou Shi Bao Yuan*
Yiqi Yangrong decoction	Middle	*You Ke Zheng ZhiZhun Sheng*
Qianghuo decoction	Middle	*Sheng Ji Zong Lu*
Zhufu decoction	Middle	*Wen Yin Ju Hai Shang Xian Fang*
Liuwei Dihuang pill	Middle	*Wai Ke Zheng Zhi Quan Shu*
Bawei pill	Middle	*Wai Ke Zheng Zhi Quan Shu*
Liuwei pill	Low	*Nv Ke Zheng ZhiZhun Sheng*
Yunmu cream	Low	*Sheng Ji Zong Lu*
Touguan powder	Low	*Sheng Ji Zong Lu*
Chuangmutong decoction	Low	*Yi Xue Zheng Zhuan*
Danggui Sini decoction	Low	*De Xin Ji Yi An*
Guipi decoction	Low	*Xu Ming Yi Lei An*
Duhuo Jisheng decoction	Low	*Wai Ke Xin Fa Yao Jue*
Wuyao Shunqi powder	Low	*Gu Jin Yi An An*
Add or subtract Wuji powder	Low	*Bing Ji Sha Zhuan*
Niuxi decoction	Low	*Sheng Ji Zong Lu*

*Note.* The table listed only the first 10 pieces of low-level evidence.

## Data Availability

Data are available upon reasonable request to the corresponding author.

## Supplementary Materials

Supplementary Materials The results of the second round of expert questionnaire and evaluation scale and the description of prevention and treatment evidence of Ancient Chinese Medicine books (V1) are shown in the supplementary files.
