# Supplementary material for: Application of the Delphi Method in the Construction of an Evaluating and Grading Scale for Evidence of Disease Prevention and Treatment in Ancient Books of Traditional Chinese Medicine
Source: Evid Based Complement Alternat Med. 2022 Mar 1;2022:3674663. doi: 10.1155/2022/3674663 (PMC8904085; doi:10.1155/2022/3674663)
Supplement: Supplementary Materials — The results of the second round of expert questionnaire and evaluation scale and the description of prevention and treatment evidence of Ancient Chinese Medicine books (V1) are shown in the supplementary files. [file 3674663.f1.docx]

| appendix 1 Results of the second round of expert questionnaire survey | | | | | | | |
| --- | --- | --- | --- | --- | --- | --- | --- |
| **Classification** | **Evaluation Index** | **Score** | **Consensus**  **Indexes** | **Handing Situation** | **Modification Situation** | **Normalized Grading Value** | **Grading Value After Rounding** |
| Evidence’s source (Ancient books) | 1. Quantity of citing others（A1） | Assigns a score according to the range of the number of retrieved items.  ＞5000: 5 points; ＞160: 3 points; ＞30: 1 point. | 86% | Pass | Nothing | 0.35 | 3.5 |
|  | 2. Quantity of version (A4) | Assigns a score according to the range of the number of versions found.  ＞50: 5 points; ＞30: 3 points; ＞5: 1 point. | 69% | Pass | Quantitative reference value:＞20:5 points; ＞10:3 points; ＞2:1 point | 0.31 | 3 |
|  | 3. Popularity of ancient books (A5) | ① Official documents and classic works: 5 points.  ② Representative works of a certain school: 4 points. ③ Literature introduced in textbooks of Traditional Chinese Medicine (except the above): 3 points.  ④ Any other work by an author representing a school of thought: 2 points. ⑤ General literature of TCM：1 point. | 90% | Pass | Nothing | 0.34 | 3.5 |
| Evidence of knowledge | 1. Is the description of disease treatment comprehensive? (A6) | Comprehensive: 5 points;  Basically comprehensive: 3 points; incomprehensive:1 point. | 86% | Pass | Nothing | 0.26 | 2.5 |
|  | 2.Are they extensively studied in other ancient medical books of knowledge? (A7) | Assigns a score according to the range of the number of retrieved items.  ＞1300: 5 points; ＞500: 3 points; ＞10: 1 point. | 79% | Pass | Nothing | 0.24 | 2.5 |
|  | 3.Is it widely used in medical cases and notes? (A8) | Assigns a score according to the range of the number of retrieved items.  ＞250: 5 points; ＞50: 3 points; ＞2: 1 point. | 83% | Pass | Nothing | 0.25 | 2.5 |
|  | 4.Is it widely used in modern literature? (A9) | Assigns a score according to the range of the number of retrieved items.  ＞30000:5 points; ＞6000:3 points; ＞750:1 point. | 72% | Pass | Nothing | 0.24 | 2.5 |
| Evidence of case | 1.Is diagnosis and treatment information comprehensive? (A11) | Comprehensive: 5 points; Basically comprehensive: 3 points; incomprehensive: 1 point. | 90% | Pass | Nothing | 0.21 | 2 |
|  | 2. Is the number of visits for disease treatment reported? (A12) | Yes: 5 points;  No: 0 point. | 86% | Pass | Nothing | 0.19 | 2 |
|  | 3. Is efficacy reported? (A13) | effective: 5 points; little change in the disease: 3 points; Ineffective, aggravating or unreported: 0 point. | 76% | Pass | Nothing | 0.21 | 2 |
|  | 4. Notes or explanation on diagnosis and treatment gist and thinking? (A16) | Describe in detail: 5 points; describe in rough: 3 points; No reference or guidance for treatment or research, or no explanatory notes: 0 point. | 86% | Pass | Nothing | 0.20 | 2 |
|  | 5. Is it widely used in modern literature? (A17) | Assigns a score according to the range of the number of retrieved items.  ＞30000:5 points; ＞6000:3 points; ＞750:1 point. | 79% | Pass | Nothing | 0.19 | 2 |

appendix 2 Evaluation scale of prevention and treatment evidence of Ancient Chinese Medicine books (V1) and description

| **Classifications** | | **Terms** | **Score** | **Grading Value** |
| --- | --- | --- | --- | --- |
| **1.** **Evaluation index of ancient books of evidence source** | | **1. Quantity of citing others** | **Assigns a score according to the range of the number of retrieved items.**  **＞5000:5 points; ＞160:3 points; ＞30:1 point.** | **3.5** |
|  |  | **2.** **Quantity of version** | **Assigns a score according to the range of the number of versions found.**  **＞20:5 points; ＞10:3 points; ＞2:1 point** | **3** |
|  |  | **3. Popularity of ancient books** | **① Official documents and classic works: 5 points.**  **② Representative works of a certain school: 4 points.**  **③ Literature introduced in textbooks of TCM (except the above): 3 points.**  **④ Any other work by an author representing academic thought: 2 points.**  **⑤ General literature of TCM：1 point.** | **3.5** |
| **2. Evaluation index of evidence content** | **Evidence of knowledge** | **1.** **Whether the description of disease treatment is comprehensive?** | **Comprehensive: 5 points; Basically comprehensive: 3 points; Incomprehensive: 1 point.** | **2.5** |
|  |  | **2.** **Whether it has been extensively studied in other ancient books of knowledge?** | **Assigns a score according to the range of the number of retrieved items.**  **＞1300:5 points; ＞500:3 points; ＞10:1 point.** | **2.5** |
|  |  | **3.** **Whether it has been widely used in ancient books of cases?** | **Assigns a score according to the range of the number of retrieved items.**  **＞250:5 points; ＞50:3 points; ＞2:1 point.** | **2.5** |
|  |  | **4.Whether it is widely used in modern literature?** | **Assigns a score according to the range of the number of retrieved items.**  **＞30000:5 points; ＞6000:3 points; ＞750:1 point.** | **2.5** |
|  | **Evidence of case** | **1. Is the diagnosis and treatment information comprehensive?** | **Comprehensive: 5 points; Basically comprehensive: 3 points; Incomprehensive: 1 point.** | **2** |
|  |  | **2.** **Whether the curative effect was reported?** | **Effective: 5 points; Little change in the disease: 3 points; Ineffective, aggravating or unreported: 0 point.** | **2** |
|  |  | **3.** **Whether reported the number of visits for disease treatment?** | **Yes: 5 points; No: 0 point.** | **2** |
|  |  | **4.** **Clarify the basis and ideas of diagnosis and treatment?** | **Describe in detail: 5 points; Describe in rough: 3 points; No reference or guidance for treatment or research, or no explanatory notes: 0 point** | **2** |
|  |  | **5.** **Whether it is widely used in modern literature?** | **Assigns a score according to the range of the number of retrieved items. ＞30000:5 points; ＞6000:3 points; ＞750:1 point.** | **2** |

| **Evaluation Index** | **Description of scoring standard** |
| --- | --- |
| **1.** **Quantity of citing others** | **It is similar to evaluating the citation rate of modern journals. The more the content is cited, the higher its importance is.** In the evaluation, the ancient book name was used as the key word, which was searched in *Zhong Hua Yi Dian*. After excluding the number of articles in the original work, the scores were graded according to the following criteria. This standard is based on the 10 pieces of evidence preprocessed, because the results are quite different, that is, there is extreme value, the median 160 is taken as the middle value and the maximum value of 5000 and the minimum value of 30 are taken for subsection scoring. Reaching the maximum value is 5 points, the median value is 3 points, and the minimum value is 1 point.  **＞5000:5 points; ＞160:3 points; ＞30:1 point.** |
| **2.** **Quantity of version** | **The more versions issued, the more widely spread, the greater the impact on future generations, so the higher the degree of importance.** General catalogue of ancient books of Chinese Medicine is used as the retrieval tool. This standard is based on the 10 pieces of evidence preprocessed, because the results are quite different, that is, there is extreme value, so the median value of 30, the maximum value of 50 and the minimum value of 5 are taken for subsection scoring. Reaching the maximum value is 5 points, the median value is 3 points, and the minimum value is 1 point.  **＞20:5 points; ＞10:3 points; ＞2:1 point** |
| **3.** **Popularity of ancient books** | The evaluation of the popularity of ancient books is based on the contents in *History of Chinese Medicine* (Reference 17). |
| **1.** **Whether the description of disease treatment is comprehensive?** | **A complete and comprehensive discussion can make the ancient book evidence users get the overall ideas and measures of treating disease.**  5 points: the main and concurrent symptoms of the diseases treated or prevented are described  3 points: including at least one major symptom and one concurrent symptom  1 point: includes at least one major symptom |
| **2.** **Whether it has been extensively studied in other ancient books of knowledge?** | **The studies in other ancient books of knowledge can reflect the importance of its content to a certain extent.** In the evaluation, prescription name (including alias) was used as the key word, which was searched in *Zhong Hua Yi Dian*. After excluding the number of articles in the original work and ancient books of medical cases, the scores were graded according to the following criteria. This standard is based on the 10 pieces of evidence preprocessed, because the results are quite different, that is, there is extreme value, so the median value of 500, the maximum value of 1300 and the minimum value of 10 are taken for subsection scoring. Reaching the maximum value is 5 points, the median value is 3 points, and the minimum value is 1 point.  **＞1300:5 points; ＞500:3 points; ＞10:1 point.** |
| **3.** **Whether it has been widely used in ancient books of cases?** | **The application of evidence of knowledge in ancient books of cases can reflect the importance of its content.** In the evaluation, prescription name (including alias) was used as the key word, which was searched in *Zhong Hua Yi Dian*. After excluding the number of articles in the original work and ancient books of medical cases, the scores were graded according to the following criteria. This standard is based on the 10 pieces of evidence preprocessed, because the results are quite different, that is, there is extreme value, so the median value of 50, the maximum value of 250 and the minimum value of 2 are taken for subsection scoring. Reaching the maximum value is 5 points, the median value is 3 points, and the minimum value is 1 point.  **＞250:5 points; ＞50:3 points; ＞2:1 point.** |
| **4.** **Whether it is widely used in modern literature?** | **The fact that the evidence in ancient books is studied in modern literature can also reflect the importance of its content.** In the evaluation, the prescription name (including alias) was used as the search term, and was searched in CNKI, Wanfang, CBM and PubMed. The number of items retrieved was graded according to the following criteria. This standard is based on the 10 pieces of evidence preprocessed, because the results are quite different, that is, there is extreme value, so the median value of 6000, the maximum value of 30000 and the minimum value of 750 are taken for subsection scoring. Reaching the maximum value is 5 points, the median value is 3 points, and the minimum value is 1 point.  **＞30000:5 points; ＞6000:3 points; ＞750:1.** |
| **1. Is the diagnosis and treatment information comprehensive?** | **The information of diagnosis and treatment mainly includes the information of look, listen, question and feel the pulse, the prescription and dosage prescribed, and the methods of decocting and taking.**  5 points: the above diagnosis and treatment information is comprehensive  3 points: the above diagnosis and treatment information is relatively comprehensive, which must include main information to help diagnosis and the information of prescriptions and doses of each medicine  1 point: at least including prescription and dose information |
| **2.** **Whether the curative effect was reported?** | **The quality of the efficacy is directly related to whether the evidence is applied.**  5 points: effective  3 points: little change in the disease  0 point: ineffective, aggravating or unreported |
| **3.** **Whether reported the number of visits for disease treatment?** | **The description of diagnosis times can make the content of medical cases more detailed and reflect the dynamic process of improvement or deterioration of the disease.**  5 points: as long as diagnosis times can be reported, regardless of the number of times  0 point: there is no record of diagnosis times, especially for the more serious cases |
| **4.** **Clarify the basis and ideas of diagnosis and treatment?** | **The more detailed the description of this article, the greater the guiding role for clinical or scientific research personnel, and the higher the degree of importance.**  5 points: it describes the etiology, pathogenesis, diagnosis and treatment ideas as process and principles of the disease in detail  3 points: describe in rough  0 point: there is no reference or guidance value for treatment or research, or there is no explanatory note |
| **5.** **Whether it is widely used in modern literature?** | **The fact that the evidence in ancient books is studied in modern literature can also reflect the importance of its content.** In the evaluation, the prescription name (including alias) was used as the search term, and was searched in CNKI, Wanfang, CBM and PubMed. The number of items retrieved was graded according to the following criteria. This standard is based on the 10 pieces of evidence preprocessed, because the results are quite different, that is, there is extreme value, so the median value of 6000, the maximum value of 30000 and the minimum value of 750 are taken for subsection scoring. Reaching the maximum value is 5 points, the median value is 3 points, and the minimum value is 1 point.  **＞30000:5 points; ＞6000:3 points; ＞750:1.** |
| **Note: the grading value of ancient book evaluation and evidence content evaluation is 3:7.**  **Standard of evidence quality classification:**  **35 points and above is high-grade evidence;**  **20 points and above is medium-grade evidence;**  **below 20 points are low-grade evidence.**  **Note: For a prescription of the same treatment measure, if it is used as knowledge evidence and case evidence, the prescription will be upgraded to a higher level based on the original knowledge evidence classification result, and it will not be evaluated in case evidence; for the same case type of evidence, first all the evidence will be included, and finally the highest level of evidence will be selected.** | |
